# Fingerprinting snakes: paleontological and paleoecological implications of zygantral growth rings in Serpentes

**DOI:** 10.7717/peerj.4819

**Published:** 2018-05-25

**Authors:** Holger Petermann, Jacques A. Gauthier

**Affiliations:** 1 Department of Geology and Geophysics, Yale University, New Haven, CT, United States of America; 2 Yale Peabody Museum of Natural History, Yale University, New Haven, CT, United States of America

**Keywords:** Skeletochronology, Squamata, Zygantrum, Growth rings, Histology

## Abstract

We introduce a new non-destructive source of skeletochronological data with applications to species identification, associating disarticulated remains, assessing minimum number of individuals (MNI), and collection management of fossil snakes, but with potential implications for all bony vertebrates, extinct or extant. Study of a diverse sample of Recent henophidian snakes confirms that annual growth cycles (AGCs) visible on the surface of the vertebral zygantrum correspond to lines of arrested growth in osteohistological thin sections and accordingly reflect chronological age. None of the specimens considered here showed signs of remodelling of the zygantrum, suggesting that a complete, unaltered age record is preserved. We tested potential influences on AGCs with a single experimental organism, a male *Bogertophis subocularis*, that was raised at a controlled temperature and with constant access to mice and water. The conditions in which this individual was maintained, including that it had yet to live through a full reproductive cycle, enabled us to determine that its AGCs reflect only the annual solar cycle, and neither temperature, nor resource availability, nor energy diversion to gametogenesis could explain that it still exhibited lines of arrested growth. Moreover, growth lines in this specimen are deposited toward the end of the growth season in the fall, and not in the winter, during which this individual continued to feed and grow, even though this mid-latitude species would normally be hibernating and not growing. This suggests that growth lines are not caused by hibernation, but reflect the onset of a physiological cycle preparing *Bogertophis subocularis* for winter rest. That being said, hibernation and reproductive cycle could still influence the amount of time represented by an individual growth line. Growth-line number and AGC spacing-pattern, plus centrum length, are used to estimate MNI of the Early Eocene fossil snake *Boavus occidentalis* collected from the Willwood Formation over two field seasons during the late 19th century. We identified eight or nine individuals among specimens previously parcelled among two specimen lots collected during those expeditions.

## Introduction

### Motivation

The vertebrate skeleton provides a rich source of data on important evolutionary and ecological parameters including climate change, seasonality, population structure, chronological age, sexual maturity, growth rate, and maximum size (Ruscillo, 2015). Vertebrates in general and “cold-blooded” poikilotherms in particular can show considerable plasticity in growth and development in response to changing environmental conditions (Lance, 2003; Petermann, Mongiardino Koch & Gauthier, 2017; Sand, Cederlund & Danell, 1995; Shine & Charnov, 1992). This can render some of these ecological parameters difficult to assess from bones alone, especially in fossils. To complicate matters further, histological study requires destructive sampling, which typically renders specimens less useful for future studies. Skeletal elements most often thin-sectioned are limb long bones, particularly the femur, tibia, and humerus (e.g., Castanet, 1994; Castanet et al., 2000). Ribs have proven a reliable alternative to long bones; they are far more numerous and bear less weight, as a consequence suffer less remodelling with age, and often preserve more complete growth records (Erickson, 2005; Erickson et al., 2004; Waskow & Mateus, 2017; Waskow & Sander, 2014). Destructive sampling can conflict with the goals of museum staff responsible for the care of rare or irreplaceable specimens. There is thus a premium on developing methods enabling less-destructive access to important life-history data from vertebrate skeletons.

Neither long bones nor ribs might serve as adequate means for estimating chronological age in limb-reduced vertebrates. In snakes, for example, long bones are either too small (e.g., scolecophidian snakes; Gauthier et al., 2012) or absent (e.g., caenophidian snakes; Gauthier et al., 2012). Except in those rare instances in the fossil record where they are found in articulation or associated with skeletons, isolated (weight-bearing) ribs often display too little morphological variation to permit assignment to taxa less inclusive than Serpentes.

**Figure 1:**
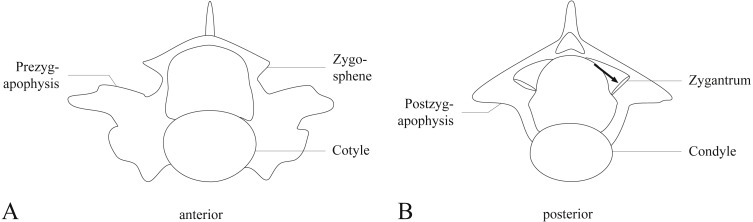
Anterior and posterior view of a pre-lymphapophyseal vertebra of *Bogertophis subocularis*. The arrow in posterior view shows the angle from which all images in Figs. 3 and 4 are shown.

Previously, age in snakes has been estimated by counting lines of arrested growth (LAGs) in thin sections of vertebral centra and ectopterygoid bones from the skull (e.g., Castanet & Naulleau, 1974; Collins & Rodda, 1994; Minakami, 1979; Waye & Gregory, 1998). While there are data indicating that growth rings in snake cranial bones correlate well with size (Bryuzgin, 1939), correlation with age is less clear (Griffiths, 1961; Warren, 1963). Warren (1963), for example, showed that macroscopically visible lines on snake zygapophyses correspond to alternating LAGs (which he called annuli) and growth zones in thin section, and suggested that their number correlates with the age of the individual. Unfortunately, the only known-age snake in Warren’s (1963) study was a prenatal Prairie Rattlesnake (*Crotalus viridis*), so he relied mainly on indirect methods to assess age based on size (an approach that has been criticised in squamates; Petermann, Mongiardino Koch & Gauthier (2017)). We propose an alternative method—using evidence for incremental growth readily apparent on the surface of the zygantrum of snake vertebrae (see below and Fig. 1).

Erickson (2003) studied the vertebral cotyle in the Bengal Monitor (*Varanus bengalensis*) and showed a correlation between the number of visible lines (described as pairs of grooves and ridges bounding growth zones) and known age of the animals, but mentioned that growth records in older individuals were more difficult to assess. This was because cartilage obscured these lines to varying degrees (Erickson, 2003). In order to investigate whether the cotyle can be used to reliably assess chronological age in snakes, we explored the usefulness of cotylar lines on the best-known life history in our sample (*Bogertophis subocularis*). Data from the zygantrum and cotyle can be obtained non-destructively (although the skeleton must be disarticulated to permit observation). To circumvent disarticulation of museum specimens, scanning technologies, such as conventional and synchrotron computed microtomography, which have previously been used for similar purposes (e.g., Konietzko-Meier & Schmitt, 2014; Sanchez et al., 2016), and could potentially be used in future analyses.

Based on life-history data obtained from Recent snakes, we addressed the following questions: (1) Do vertebral growth marks yield a reliable age estimate for snakes; (2) what time of year do growth lines occur, and what time-spans might they reflect; and (3), can vertebral growth marks be used to identify (i.e., ‘fingerprint’) isolated fossil remains of *Boavus occidentalis* at the level of individual organisms? In order to address these questions, it is necessary to first discuss known influences on bone growth, and introduce the osteological feature this study is focused on.

### Influences on growth

Bone growth is appositional, i.e., bone mineral deposition occurs onto existing surfaces (Hall, 2005). Apposition happens mostly on the outer (periosteal ossification), but also on the inner bone surfaces (endosteal ossification) (Francillon-Vieillot et al., 1990). There are two alternating phases of bone deposition visible in long-bone shafts (Francillon-Vieillot et al., 1990); a phase of accelerated bone deposition, the growth zone (Francillon-Vieillot et al., 1990), followed by a phase in which bone deposition is very slow or stops altogether (Francillon-Vieillot et al., 1990). Reduction in bone deposition results in an annulus (*sensu* Francillon-Vieillot et al., 1990), whereas cessation in bone deposition results in a line of arrested growth (LAG; Francillon-Vieillot et al., 1990). The capacity of vertebrates to record annual growth cycles evolved at least 445 Ma (Late Ordovician) as LAGs and annuli are apparent as soon as mineralized tissues evolved (Keating, Marquart & Donoghue, 2015).

Bone-deposition rates are generally linked to extrinsic and intrinsic factors. Extrinsic factors include circadian and circannual cycles in solar insolation, abundance of food resources, climate change, inter- and intraspecific competition, and natural disasters (e.g., Lance, 2003; Nylin & Gotthard, 1998; Sand, Cederlund & Danell, 1995; Shine & Charnov, 1992). Intrinsic factors include physiology (biological clocks, metabolic rates, hormone levels, and reproductive cycles) and behavior (activity levels, territorial defence, habitat preferences) (e.g., Cubo et al., 2008; Nylin & Gotthard, 1998; Shine & Charnov, 1992). For example, Köhler et al. (2012) linked LAGs and fibrolamellar bone tissue in the periosteum to time of death to constrain the time interval during which growth might stop in ruminants for up to three or four months and found this period to coincide with harsher environmental conditions (drought or low precipitation). Köhler et al. (2012) then correlated ruminant growth with Köppen-Geiger climate zones, arguing further that hormonal activity is an important aspect in bone growth cyclicity. Of these extrinsic and intrinsic factors LAG- and annulus-thickness and/or duration is potentially influenced by seasonal cycles in resource abundance, which in turn result from annual changes in weather (Esteban, García-París & Castanet, 1996; Petermann, Mongiardino Koch & Gauthier, 2017; Smirina, 1994) and day length (Castanet et al., 2004). Conversely, growth-zone thickness likely depends on growth hormones, food abundance, and temperature and precipitation (De Margerie, Cubo & Castanet, 2002; Petermann, Mongiardino Koch & Gauthier, 2017). In addition, there is evidence for a genetically determined rate of bone deposition governing the amount and type of bone tissue laid down (Price et al., 2005), which could influence growth-zone thickness. While there is evidence that hormone levels affect bone growth, the underlying causal relations in, for example, exactly how, and for how long, bone deposition is interrupted to produce a LAG, annulus, or other type of growth line, needs further investigation.

### The zygantrum

**Figure 2:**
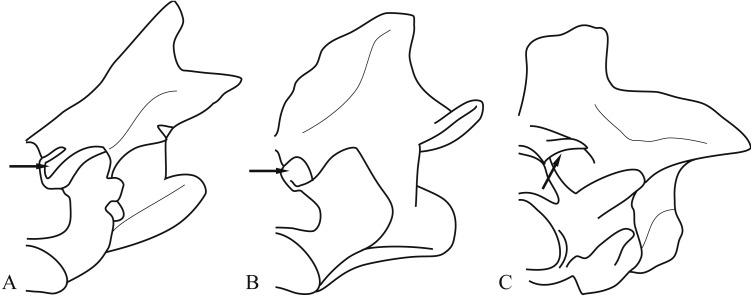
Geometries of zygosphene-zygantrum intervertebral articulations (description in text). (A) *Trachylepis quinquetaeniata*. (B) *Gambelia wislizenii*. (C) *Tropidophis haetianus*. All vertebrae in oblique anterior view. Arrows point at zygosphene-zygantrum articulation surface. Modified from Gauthier et al. (2012).

The lepidosaur accessory intervertebral joint consists of the zygantrum and the zygosphene situated between the primary intra-neural arch joint formed by the zygapophysis. Zygantra are a set of two distinct facets located posteriorly on the vertebra, dorsal to the neural canal and dorsomedial to the postzygapophysial facets, and lying between the neural arch and postzygapophysis (Fig. 1). Zygosphenes, the other component of this joint, are a set of distinct facets located anteriorly on the vertebra, dorsal to the neural canal (in snakes), and between the neural arch and prezygapophyseal facet with which it was originally continuous. Gauthier et al. (2012) identify three states for this accessory joint: (1) the zygosphene facet is continuous with the prezygapophyseal facet on the margin of the neural arch and faces dorsolaterally (Fig. 2A; Gauthier, Estes & De Queiroz, 1988; Gauthier et al., 2012); (2) the zygosphene facet is continuous with the prezygapophyseal facet, but is produced dorsal to the neural canal’s apex and faces more laterally (Fig. 2B; Gauthier et al., 2012); and (3) the zygosphene facet is separate from the prezygapophyseal facet, is set on a distinct pedicle, and faces ventrolaterally (Fig. 2C; De Queiroz, 1987; Estes, De Queiroz & Gauthier, 1988; Gauthier et al., 2012). This accessory intervertebral joint has been thought to stabilize the vertebral column and reduce torsion along the long axis of the vertebral column ((Romer, 1976); but see Moon (1999) who shows that at least some torsion is still possible between successive vertebrae). The zygosphene-zygantrum joint exhibits varying degrees of development, including secondary loss, among major clades of lepidosaurs (Evans, 1981; Gauthier, Estes & De Queiroz, 1988; Gauthier et al., 2012).

Apparently non-homologous accessory intervertebral articulations have been observed in other reptilian clades, for example, in the younginiform *Hovasaurus boulei* (Currie, 1981), and in some archosaurs such as the rauisuchid *Batrachotomus kupferzellensis* (Gower, 1999), the silesaurid *Silesaurus opolensis* (Piechowski & Dzik, 2010) and, most notably, in the hyposphene-hypantrum accessary joint diagnostic of saurischian dinosaurs (Gauthier, 1986). Within Saurischia, the hyposphene-hypantrum is lost secondarily in Aves (Gauthier, 1986), and in titanosaur and rebbachisaur sauropods (Apesteguía, 2005). The saurischian accessory joint features a distinct process, the hyposphene, located posteriorly on the vertebra that is ventral to, and continuous with, the postzygapophysis (Apesteguía, 2005). The hyposphene fits into a slot, the hypantrum, formed by the close-set prezygapophyses of the following vertebra, and is expressed mostly in dorsal vertebrae (Apesteguía, 2005). As in lepidosaurs, details of joint morphology vary (for description see Apesteguía, 2005; Stefanic & Nesbitt, 2018), and a hyposphene-hypantrum joint has been suggested to improve stability of the vertebral column (Fronimos & Wilson, 2017).

## Materials and Methods

### Taxa studied

We studied the pre-lymphapophyseal vertebrae of six henophidian snakes (Table 1): the pythonid *Morelia spilota* LaCépède, 1804 (Carpet Python), the boid *Candoia aspera* Günther, 1877 (New Guinea Ground Boa), the viperid *Bitis rhinoceros* Schlegel, 1855 (West African Gaboon Viper), and the three colubrids *Rhabdophis tigrinus* Boie, 1826 (Tiger Keelback), *Euprepiophis mandarinus* Cantor, 1842 (Mandarin Rat Snake), and *Bogertophis subocularis* Brown, 1901 (Trans-Pecos Rat Snake). *Morelia spilota* was donated to the YPM collections by a private breeder, Brian Kleinman; *Bogertophis subocularis* was donated by another breeder, Gordon Schuett; the remaining specimens were sent to Yale from Sedgwick County Zoo, Kansas. These taxa effectively bracket the ancestral henophidian (*sensu* Gauthier et al., 2012; Hsiang et al., 2015), if not all Serpentes.

**Table 1:** Number of LAGs visible on vertebral zygantra of *Bogertophis subocularis*.

	Collection number (YPM R)	SVL (cm)	Date of birth	Date of death	Age (years)	Growth lines	Left zygantrum		Right zygantrum		Combined		Mean centrum length (mm)	*σ* (mm)	*σ* (%)
			(dd/mm/yyyy)				#	%	#	%	#	%			
*Morelia spilota*	19419	191.2	before 2005 (acquired at 8–10 years)	2015	18–20	17	247	92	240	89	261	97	9.16	1.79	19.5
						18	21	8	29	11	8	3			
*Candoia aspera*	22001	45.8	before 01∕01∕2012	14∕01∕2016	at least 4	4	10	7	7	5	4	3	3.4	0.43	12.7
						5	131	93	133	95	137	97			
*Bitis rhinoceros*	19191	184.7	between 01∕12∕2007 and 01∕12∕2008	12∕05∕2014	5.5 to 6.5	5	18	14	24	18	7	5	9.41	1.83	19.5
						6	113	86	107	82	124	95			
*Rhabdophis tigrinus*	19193	81.5	estimated 27∕03∕2010	12∕05∕2014	listed as 4	3	6	4	11	7	4	3	5.78	0.56	9.7
						4	150	96	146	93	153	97			
*Euprepiophis mandarinus*	20688	102.5	28∕07∕2007	15∕02∕2017	6.5	5	22	10	20	9	6	3	3.99	0.4	10
						6	200	90	202	91	216	97			
*Bogertophis subocularis*	19102	66	fall 2011	23∕09∕2013	2	1	22	9	18	8	5	2	2.52	0.26	10.1
						2	215	91	220	92	233	98			

**Figure 3:**
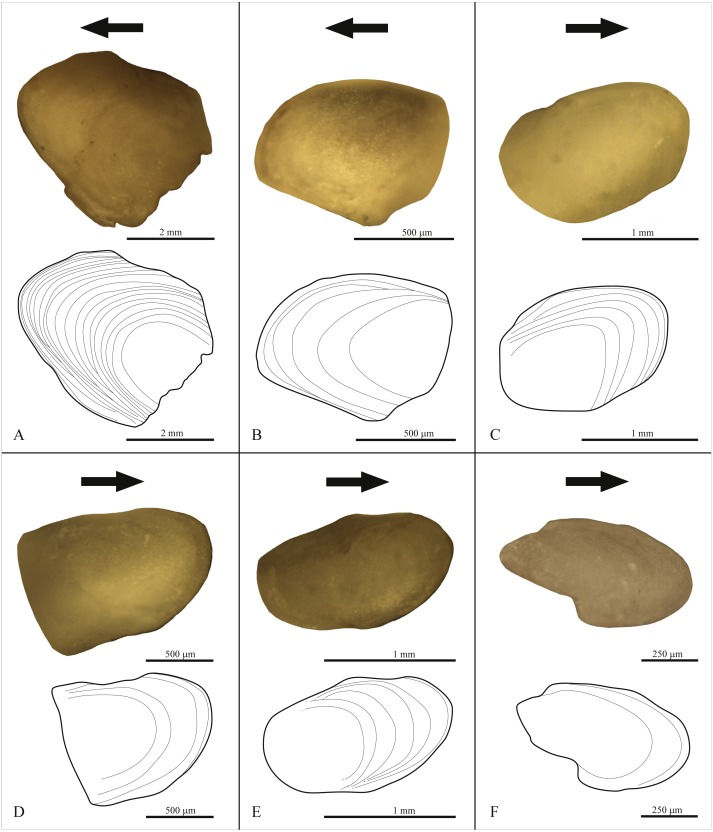
Oblique dorsal view of one zygantrum for each studied specimen. (A) *Morelia spilota*, 18 growth lines. (B) *Candoia aspera*, five growth lines. (C) *Bitis rhinoceros*, six growth lines. (D) *Rhabdophis tigrinus*, four growth lines. (E) *Euprepiophis mandarinus*, 6 growth lines. (F) *Bogertophis subocularis*, 2 growth lines. Arrows point posteriorly.

**Figure 4:**
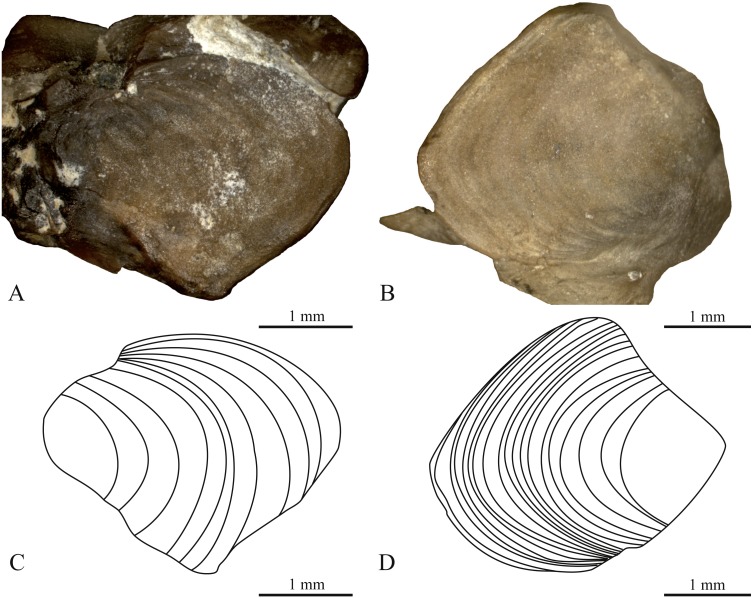
Zygantra of *Boavus occidentalis* from the Early Eocene of Wyoming in oblique dorsal view. (A, C) YPM VP 59192; 11 AGCs. A vertebra associated with YPM VP 2770, generally dark brown in colour. (B, D) YPM VP 59197; 19 AGCs. A vertebra associated with YPM VP 3752, lighter in colour than vertebrae assigned to YPM VP 2770.

We recorded the number of annual growth cycles by counting distinctive lines (LAGs) on the zygantrum (Figs. 3 and 4) under the microscope (see details below). We further recorded relative growth among successive annual growth cycles (AGCs) (i.e., the spacing and distinctiveness of successive growth lines; Figs. 3 and 4). Yale University’s Institutional Animal Care and Use Committee provided full approval for this research (IACUC-#: 2015-10681; protocol PI: Prof. Thomas J. Near).

The only specimen for which we had complete data was *Bogertophis subocularis* (YPM R 19102). The individual was born into captivity; the breeding colony was established with adults taken in Brewster Co., Texas, in the 1980s. In order to remove all potential external influences on growth apart from photoperiod, it was raised in a stable environment for two years (Fall 2011 to Sept. 2013) at a temperature between 26° and 28 °C and regularly fed and watered throughout the year. It was exposed only to light naturally available through the window on daily and annual cycles available at ∼34°N. The individual was not forced into winter rest, and was active year round (G Schuett, pers. comm., 2017). When euthanized, the specimen was 77 cm long (total length = TL; snout-vent length, SVL = 66 cm), which is about 52% to 68% of the typical TL (114–147 cm) recorded for male *B. subocularis* (Rhoads, 2008). According to Rhoads (2008), age of the specimen places it at the minimum for sexual maturity (which he reports as 18 to 24 months based on observations in captive individuals). Price (2006) reported that size at sexual maturity for captive male *B. subocularis* is 76 cm TL, virtually the same size as our experimental specimen, which means that it had yet to undergo spermatogenesis. Age at sexual maturity in wild snakes is rarely known, and *B. subocularis* is no exception.

We knew date of birth and date of death for *Euprepiophis mandarinus* (Table 1). Our specimen was within the typical adult size reported for this species (Table 1), which can range from 100 to >170 cm TL (Gumprecht, 2004; Schulz & Münzenmaier, 1990). Sexual maturity is listed as 3–4 years and 90 cm TL (Gumprecht, 2004; Schulz & Münzenmaier, 1990), both of which are exceeded by our specimen (Table 1). The other snakes from Sedgwick County Zoo had associated dates of death, but inexact entries for date of birth (Table 1). This resulted in age ranges (*Bitis rhinoceros*; Table 1) and minimum age estimates (*Candoia aspera* and *Rhabdophis tigrinus*; Table 1). The *B. rhinoceros* was in the range of adults (Table 1), which is listed as beginning at 130 cm TL and an age of three according to Marsh & Whaler (1984). Harlow & Shine (1992) report a strong sexual dimorphism in adult SVL for male and female *C. aspera*, and our male specimen is slightly longer than the average adult male (Table 1). Unfortunately, age at which sexual maturity is achieved is not known for any *Candoia* (Harlow & Shine, 1992). The *Rhabdophis tigrinus* exhibits sexual dimorphism; females, such as ours, typically reach adult size of 78.5–101 cm SVL (Tanaka & Ota, 2002), placing our specimen among the small adults (Table 1). As with *Candoia*, age at sexual maturity is not available for wild *R. tigrinus*. We had no information on the conditions any of the animals from Sedgwick County Zoo were raised under (temperature, light cycles, induced hibernation, etc.) or the cause of death. Similarly, we had only scant knowledge of the life history of *Morelia spilota*; the breeder acquired the specimen as a sexually mature adult (Table 1). No information was available about the conditions in which it was kept. Adult male *M. spilota* are reported to range from 143–182 cm SVL, reaching sexual maturity at 150 cm SVL (Slip & Shine, 1988). Our male specimen is an exceptionally large and old *M. spilota* (Table 1). Data on age at sexual maturity was not available for this species.

After removing skin and viscera, we excised the tongue and hyoid apparatus, and preserved these soft tissues separately in an ultracold freezer. The skeleton was then dried beneath a fume hood and defleshed by dermestid beetle larvae. Trunk vertebrae were strung together with monofilament line and disarticulated by maceration for three weeks in water taken from a local river. We used all trunk vertebrae but typically the first few vertebrae could not be detached from the skull and those vertebrae were excluded from consideration (see Table 1 for number of vertebrae). We examined the zygantra on both sides on all vertebrae for growth lines reflecting AGCs for all specimens (including fossil *Boavus occidentalis*; see below). These lines typically manifest as grooves set between ridges. The position of the zygosphene, obscured by being in opposition to and nearly parallel with, the prezygapophysis, makes it nearly impossible to investigate with light microscopy, which is why we decided against the inclusion of this feature for comparison to the zygantrum. The orientation of the zygantrum aligns with the growth axis of the postzygapophysis (Fig. 1), thereby reducing the likelihood of subsequent LAG resorption with growth, as is often the case in long bones of aged vertebrates.

*Boavus occidentalis* Marsh, 1871 is represented in this study by 12 isolated vertebrae from the Willwood Formation in Wyoming, parcelled between two specimen numbers (eight vertebrae to YPM VP 2770, now YPM VP 59189-193, and four vertebrae to YPM VP 3752, now YPM VP 59194-197; Table 2). Although we cannot determine if these vertebrae belong to the boid crown clade, their stocky build, neural-spine form, and details of intervertebral joint anatomy firmly place them among the boid total group (= Pan-Boidae), and thus part of Booidea, a clade that originated during the Late Cretaceous (Hsiang et al., 2015). The specimens are described as having been found at the same locality by two separate collectors in 1870 and 1872, and include many fragmentary vertebrae in addition to the 12 considered here. Following the method outlined above for Recent snakes, we used growth records preserved on the vertebrae to determine age-in-years for each and thereby assess whether they represented the remains of the same or different individual snakes. We also measured centrum length for size comparison of fossil specimens with the same number of AGCs, and compared the results to size variation among precloacal vertebrae in *Morelia spilota* and *Candoia aspera*, disparate snake species that bracket phylogenetically the ancestral booid.

**Table 2:** Growth in *Bogertophis subocularis* calculated from size increase between successive LAGs.

Original YPM VP	New YPM VP	LAGs	Centrum length (mm)	Individual count
2770	59189	6	8.8	1
	59190	8	10.41	2
	59190	8	10.91	2
	59191	9	10.67	2, 3
	59191	9	10.98	2, 3
	59191	10	9.66	3
	59192	11		3, 4
	59193	13	10.58	5
3752	59194	10	10.63	1
	59195	12	8.61	2
	59196	14	9.76	3
	59197	19		4

### Osteohistology

**Figure 5:**
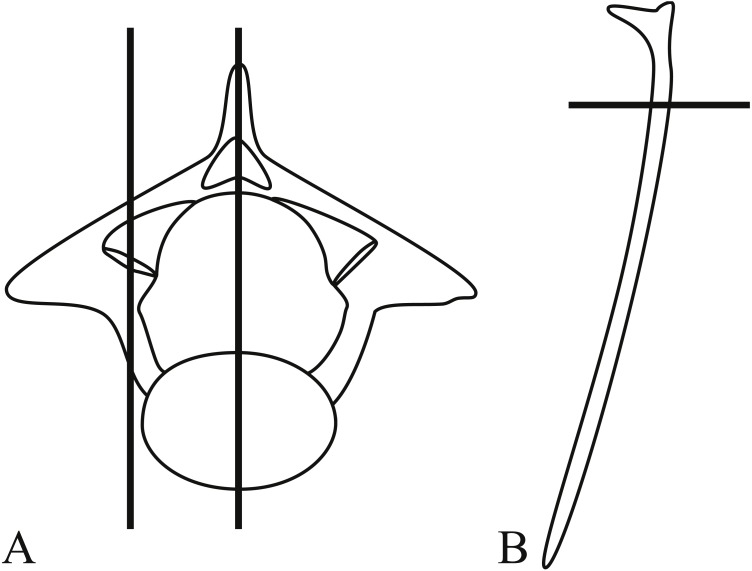
Thin-sectioning planes through the vertebrae (A) and ribs (B).

We sent two consecutive vertebrae (#131 and #132), and one of the corresponding ribs from each segment, from *Bogertophis subocularis* to Vancouver GeoTech Labs for thin-sectioning (see Fig. 5 for sectioning planes). The company largely follows the thin-sectioning protocol outlined by Petermann & Sander (2013), but differs in that the bones were vacuum embedded with Southern Polyurethanes Incorporated (SPI) Spurr-type epoxy prior to thin-sectioning. The bone wafers were mounted with SPI #1300 epoxy (both epoxies cure at room temperature). Thin sections were machine-polished to 100 µm thickness following Bromage & Werning (2013). No other chemicals were applied to the specimens prior to, during, or post-sectioning.

**Figure 6:**
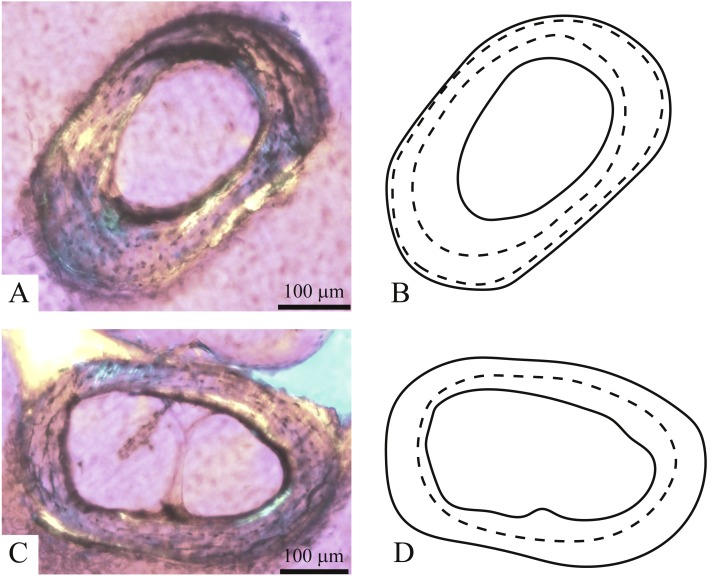
Transverse cross-sections proximal on the shaft of the ribs corresponding to vertebrae #131 (A, B) and #132 (C, D) of *Bogertophis subocularis*. The number of LAGs in the ribs corresponds to the number of seen on the zygantra of the vertebrae.

The ribs were cut transversely and proximally on the shaft (Fig. 5). The two vertebrae were cut longitudinally at an oblique angle, aiming to cut through the zygantrum in both cases. However, they only managed to cut one vertebra through the zygantrum, while the second one was cut near the midline (Figs. 5 and 6).

### Microscopy

Vertebral surfaces were examined using light microscopy on a Leica MZ 16 microscope with a Leica DFC 420 camera attachment. We documented vertebrae from the oblique dorsal aspect of the zygantra (Figs. 1, 3 and 4). An additional image was taken to measure centrum length in lateral view. Thin sections were studied on a Leica DM 2500P polarization microscope with crossed polarizers and a lambda filter, and recorded with a ProgRes CFscan camera attachment from Jenoptic. We measured the circumference of each growth line and the contour of the zygantrum in the ImageJ (Schneider, Rasband & Eliceiri, 2012), and centrum length using Leica Application Suite 2.5 (http://leica-application-suite.updatestar.com/).

## Results

### Extant example

The number of zygantral growth lines is consistent along the vertebral column, typically varying by no more than one line in very few vertebrae among all of our specimens of henophidian snakes (Table 1). Very rarely did left and right zygantra both show the divergent number on the same vertebra (2–5%, Table 1). The python *Morelia spilota* was an exception, with some vertebrae showing extensive damage to the zygantra from preparation that artificially reduced the number of growth lines (Supplemental Information). Damage to a zygantrum very rarely (one to three instances per specimen) obliterated the growth record completely, and sometimes the growth record could not be determined in vertebrae that were fused to one another (Supplemental Information). Table 1 summarizes the relevant data.

Growth-line-spacing is consistent for all vertebrae in each specimen. In the pythonid booid *Morelia spilota* the first 11 lines among 18 are wider-spaced, with spacing somewhat decreasing toward line 11 (Fig. 3A). These lines occur over ∼75% of total zygantral length. Lines 11–18 are more closely spaced (Fig. 3A). In the boid booid *Candoia aspera* the distance between subsequent lines steadily decreases over the first three lines, and the last two lines are closely spaced (Fig. 3B). Line spacing in the viperid caenophidian *Bitis rhinoceros* is typically wider for the first three lines, extending over more than half of zygantral length, whereas the outer lines are closely spaced (Fig. 3C). The colubrid caenophidian *Rhabdophis tigrinus* follows a similar pattern; the innermost two lines are spaced widely and cover about three quarters of the zygantrum, and the outer lines are more closely spaced (Fig. 3D). The inner lines in the colubrid caenophidian *Euprepiophis mandarinus* are wider-spaced, cover about 50% of zygantrum length, and are followed by more closely spaced outer lines (Fig. 3E). In the colubrid caenophidian *Bogertophis subocularis* both growth lines have standard positions along the long axis of the zygantrum in all 238 vertebrae examined (Fig. 3F). The first growth line is set at about 2/3 the length of the zygantrum, whereas the second growth line is always set close to the outer perimeter (Fig. 3F).

### Histological evidence

Thin sections cut from the ribs of *Bogertophis subocularis* showed unvascularized parallel-fibered bone (Fig. 6). One rib showed two LAGs, the inner LAG at around 2/3 radius, whereas the outer LAG was forming very close to the perimeter (Figs. 6A and 6B). The second rib showed one LAG, located at about 2/3 the radius of the cortex (Figs. 6C and 6D).

**Figure 7:**
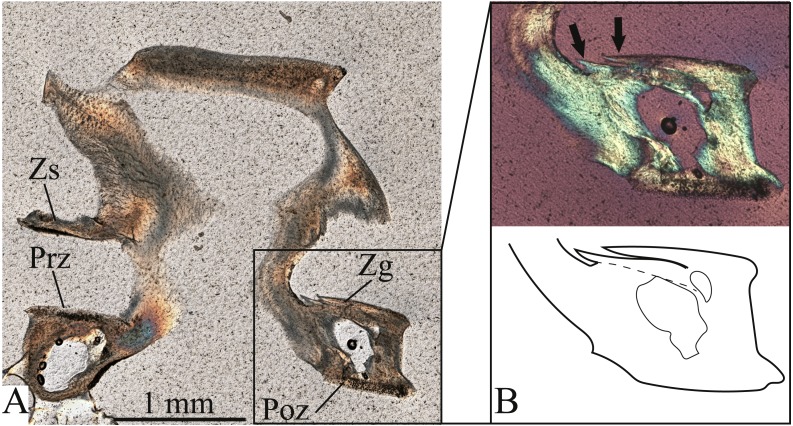
Oblique longitudinal thin section through the zygapophyses of vertebra #131 of *Bogertophis subocularis*. (A) Prz, prezygapophysis; Poz, postzygapophysis; Zs, zygosphene; Zg, zygantrum. (B) Focus view of the zygantrum, the two growth zones are visible as distinct bony sheaths (arrows) that are separated by a LAG.

**Figure 8:**
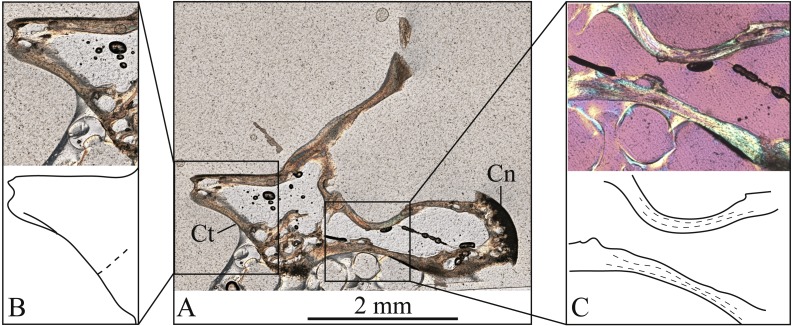
Oblique longitudinal thin section through the centrum of vertebra #132 of *Bogertophis subocularis*. (A) Cotyle (Ct) and condyle (Cn) are partially cut. (B) a LAG, continuous with a growth line visible on the cotyle (solid line), and the textural change from trabecular bone core to the glassy bone (dashed line) are visible. (C) two LAGs, corresponding to the growth-line count on the zygantrum of this vertebra, are visible in the centrum.

The vertebrae also showed unvascularized, parallel-fibered bone. Vertebra #131, cut through the zygantrum, displayed two distinct bone sheaths that are layered on top of one another, separated by what appears to be a LAG (Fig. 7). The outer margins of the two bony sheaths bend slightly upward, giving them the appearance of ridges, with the LAG appearing as a groove between them (Fig. 7). The first sheath is at about 2/3 the width of the zygantrum, whereas the second sheath forms at the outer margin (Fig. 7). This observation matches the line count for this vertebra. Because of their superficial placement, zygantra suffer no internal remodelling as they grow out from the neural arches, and therefore they preserve complete growth records, unlike long bones in other vertebrates. Thus, zygantra afford a complete record, rather than a minimum age, in lepidosaurs. The second vertebra, #132, displays two LAGs in the centrum (Fig. 8C), corresponding to the number of lines observed on this vertebra’s zygantrum, but also a line continuous with that visible on the cotylar surface (Fig. 8A).

### Extinct example

We found a varying number of growth lines on the zygantra of *Boavus occidentalis* (Table 2). If they correspond to annual growth cycles, then these vertebrae were produced by organisms that ranged in age from six to 19 years, with a median age of ten years (Table 2). YPM VP 2770 included individuals in which AGCs varied in conspicuousness, but seem to range from six to 13 years old. In contrast, AGCs in specimens included in YPM VP 3752 are easier to discern and range from 10 to 19 (Table 2; Fig. 4). We presume that discernibility reflects taphonomic differences. All zygantra investigated here are intact and their growth records are considered complete.

Different growth-line counts indicate vertebrae from different individuals, whereas relative positional relationships between AGCs and centrum length (size proxy) are useful to determine if vertebrae with the same growth-line count stem from the same individual. Standard deviation *σ* of centrum length for all species studied varied from 10% to 19.6% with the two closest extant relatives of *Boavus occidentalis* (*Morelia spilota* and *Candoia aspera*) having values of 12.7% and 19.6%, respectively (Table 1). We used these two values to set upper and lower boundaries for centrum length expected for pre-lymphapophyseal vertebrae derived from the same individual preserved in one locality.

In YPM VP 2770, five of the eight studied vertebrae had either eight (2), nine (2), or ten (1) AGCs. The vertebrae with nine AGCs were both within 2 *σ* of the centrum length of the vertebrae with eight AGCs, as well as the centrum length of the vertebra with ten AGCs. Spacing-patterns of the four vertebrae were very similar, which allowed us to group together vertebrae with the same growth-line count. Because of the overlap in size, however, we cannot associate the two vertebrae with nine AGCs with the vertebrae with eight nor the vertebra with ten AGCs unambiguously, and therefore have a count of either four or five individuals for the eight vertebrae assigned to YPM VP 2770 (Table 2).

## Discussion

### Lines on centrum cotyle are less reliable age-indicators

The most frequent morphological transition in the cotyle, present in all vertebrae of *Bogertophis subocularis*, is a change in texture from granular to glassy that corresponds to the trabecular bone core reported by Erickson (2003) and the notochord canal reported by Hoffstetter & Gasc (1969). This “line” reflects a change in bone organization in thin sections of the cotyle (Fig. 8). The second mark (present in 67%), associated with a color change in the granular portion, may reflect bleaching as result from maceration of the specimen rather than age. It could also correspond to the birth line that Erickson (2003) reported for vertebrae of Bengal Monitor (*Varanus bengalensis*). The other two circular marks are much less frequent (5.5% and 3.8%; Supplemental Information) and only occur in the glassy area. These two circular marks are continuous with LAGs visible from thin sections (Fig. 8) and thus represent growth lines, but are much less useful for age determination because of their rarity. This is in contrast to Erickson (2003), who found the cotyle to predict age accurately and reliably in the Bengal Monitor (except in older individuals; see above).

### Zygantral growth lines correspond to histological LAGs

In *Bogertophis subocularis*, LAGs revealed in thin sections through the centra accurately reflect age. Just as Warren (1963) used histology to connect internal lines with those expressed on the zygapophyses, our sections demonstrate continuity between lines visible on the zygantrum with those revealed in thin sections of the centrum (Fig. 7). Rib thin-sections also correspond with age, albeit with noticeable remodelling (Fig. 6). Remodelling of the ribs is in line with increased burden on ribs during snake locomotion due to muscles attached to various points along the rib shafts (Gray, 1946; Lissmann, 1950; Mosauer, 1932). We think that this remodelling diminishes their usefulness for accurate age determination in snakes.

### Lines in zygantrum yield accurate age assessment

We consistently found growth-line numbers along the entire vertebral column to match chronological age of the studied snakes. The few deviations in growth-line count from presumed age could be due to imprecise records for captive-bred individuals or age estimates for wild-caught individuals (e.g., for *Bitis rhinoceros*, 5.5 years ± 1 year), or to difficulty in distinguishing faint LAGs in some instances. However, we reemphasize that zygantral growth lines do not undergo resorption or remodelling, as indicated by the matching number of growth lines with age for all adult snakes including the very old *Morelia spilota* (18 years and 18 LAGs). That enables us to reconstruct life history without having to retro-calculate missing growth records. That is not the case for other skeletal elements typically used in skeletochronology, which are less reliable for age-assessment owing to frequent remodelling, especially in older individuals (examples for retro-calculation of missing LAGs: Castanet & Cheylan, 1979; Chinsamy, 1993; Horner & Padian, 2004).

It may be noteworthy that there can be some highly constrained variation in age estimates across zygantra from the same organism, albeit very rarely (2–5%) and then only by one year (Table 1). Where such variation exists, it is randomly distributed along the vertebral column, and the outliers often have one less LAG than expected (e.g., *Candoia aspera* displays five LAGs 97% of the time, but only four LAGs in 3% of its zygantra). However, the opposite case holds for *Morelia spilota*, in which 97% of its zygantra show 17 LAGs but 3% display 18 LAGs. There is no evidence for remodelling, so the source of this very modest variation remains unclear. Aside from *M. spilota* in which this datum is unknown, our snakes died at or near the onset of their annual growth cycles in the spring (Table 1) (except in *Bogertophis subocularis* that died in the fall, which was the start of a new growth cycle in this experimental animal). That is confirmed by our growth records, as the final LAGs are invariably laid down very near to the periosteum. That is to say, if the last LAG is further from the periosteum, this would indicate that it died later in the growing season, but earlier in the growing season if the last LAG is deposited closer to the periosteum (see Köhler et al., 2012).

Growth-line-spacing patterns are characteristic for each snake and seldom varied, suggesting that the overall pattern can be used to associate isolated material. Nevertheless, the exact extent and nature of spacing-pattern variability needs more thorough investigation. The general trend is similar to observations in traditional osteohistological studies, i.e., line spacing decreases with age corresponding either to sexual maturity, or osteological maturity, or both (e.g., Buffrénil & Castanet, 2000; Castanet et al., 2004; Castanet, Francillon-Vieillot & Bruce, 1996; Petermann, Mongiardino Koch & Gauthier, 2017; Sand, Cederlund & Danell, 1995; Sander, 1990; Tucker, 1997; Zug et al., 2002; Zug & Rand, 1987).

Due to the low number of individuals per species that were available for this study we could not test whether intra-specific variability affects the reliability of the method. However, we studied members from both primary branches of Henophidia and consistently found zygantral LAGs to very strongly correlate with chronological age. Following the logic of the Extant Phylogenetic Bracket (Witmer, 1995), we infer that this correlation also obtained in the most recent common ancestor of Henophidia, and thus in the extinct booid henophidian *Boavus occidentalis*. Nevertheless, we acknowledge the preliminary nature of our analysis and encourage further testing.

Application of this non-destructive method to the fossil species *Boavus occidentalis* suggests that it was a long-lived species (Table 2). It further suggests that *B. occidentalis* populations were age heterogeneous. Variability indicates that multiple individuals are represented by the two accession numbers. We used the observed variability of growth-line number from the Recent sample as a margin of error (age ± 1 year) for assessing the minimum number of specimens in the fossil sample to account for potential variability within an individual fossil snake. Presence of at least eight individuals of *B. occidentalis* in the same locality suggests that this species was a common faunal element.

### Bogertophis subocularis

The life history of *Bogertophis subocularis* is well known, as are the conditions under which our experimental animal was maintained. These factors enable us to make some additional inferences about growth and development for this individual.

Juvenile *Bogertophis subocularis* emerge from winter rest late in April and adults in May throughout their range (Rhoads, 2008; Tennant, 2006). Mating occurs in June and July, but can extend into August (Cranston, 1993); eggs are laid in late July and early August, and incubate from 75 to 90 days depending on temperature (Rhoads, 2008). Hatchlings emerge between October and December (Rhoads, 2008); our specimen hatched in Fall of 2011. Adult individuals go back into winter rest in September, and juveniles in October (Rhoads, 2008; Tennant, 2006). However, observations in the wild of umbilical scars in young *B. subocularis* emerging the year following egg deposition have given rise to speculation about hibernation in or near the nest (Rhoads, 2008), or the possibility of delayed development and hibernation while still in the egg (Tennant, 2006). Both could result in additional growth and a delayed deposition of the first growth line. Our *B. subocularis* was not forced into winter rest after hatching, but instead was kept warm, and fed and watered, continuously for its entire two-year life (G Schuett, pers. comm., 2017). This means that the growth lines visible in this specimen could not have been caused by either temperature or resource availability because these variables were fixed, which is in contrast to previous studies on growth-line deposition in snakes (e.g., Bryuzgin, 1939; Peabody, 1961).

Our *Bogertophis subocularis* just reached the age and size associated with sexual maturity (Price, 2006; Rhoads, 2008; Werler & Dixon, 2010), and therefore the primary focus of the snake’s physiology was on bone growth, so its LAGs cannot represent physiological diversion to reproduction. Annual cycles in day length are the most likely explanation for cyclicity in AGCs in this individual. This is in agreement with Castanet et al. (2004), who suggested that photoperiodicity played an important role in growth cyclicity. Given the size of the previous AGC, the date on which the specimen was euthanized in late September, and the onset of a new growth cycle, we reason that growth lines are deposited late in the normal growing season and within a limited time span (days to a few weeks at most), and not during the winter months as one might expect. Rather than hibernation causing a growth line, growth lines may instead mark onset of an intrinsic physiological slowdown in preparation for surviving the winter months. It may be noteworthy that the apparent position of the sun in the sky changes most rapidly during the fall (and spring). If *Bogertophis subocularis* could perceive these relatively rapid changes in either the quality or quantity of light during that period, this could provide the cue to the hormonal system regulating the annual growth cycle (paralleling the results in Köhler et al., 2012).

## Conclusions

From our preliminary study of a diverse sample of Recent henophidian snakes, including an experimental specimen of *Bogertophis subocularis,* we conclude that bony areas on the zygantrum separated by distinct lines correspond to annual cycles of changing rates of bone deposition, and presumably in growth rates, and thus afford a reliable assay for an individual’s age. Lack of any indication of remodelling of this superficial feature in the studied adult and senescent snakes and high consistency of number of LAGs along an individual vertebral column for all studied specimens suggest that the zygantrum is an exceptional age-correlate in snakes. We found evidence that growth-line-spacing patterns are largely consistent throughout an individual, and therefore can also be used to effectively “fingerprint” disassociated material. Nevertheless, the rarity of known-age snakes precluded investigation of potential intra-specific variability in growth-line counts, and requires further study.

As an example, our experimental *Bogertophis subocularis* vertebrae grew from the time they condensed about the notochord to their size at sexual maturity in two seasons, confirming rapid initial growth from embryo to sexual maturity. Given constant temperature and resource availability these AGCs are apparently governed by annual cycles in solar insolation, although the duration represented by LAGs could vary in response to colder temperatures and accompanying resource shortages. How diminishing day lengths are perceived (especially in a nocturnal snake), as well as how that governs the end of one AGC and the beginning of the next (marked by a LAG), are not yet understood. All vertebrae with a peripherally positioned LAG showed evidence of resumed growth shortly before this individual’s death in late September, when it normally would begin hibernation in the wild. The exact timing and timespan of growth-line production is unclear, but limited additional growth after deposition of the peripheral LAG indicates that it could have stopped for only a short period of time. Because growth lines appeared despite continuous feeding and constantly suitable temperature, a genetic component may play an important role in governing growth cycles. Together with examination of spacing-patterns among individual AGCs, and comparable size distributions within single vertebral columns, this method enables us to effectively fingerprint disarticulated vertebrae from fossil and Recent snakes, justifying assignment to the same organism. Not only did this method increase the specimen count for *Boavus occidentalis* from an Early Eocene locality in Wyoming, it also offers a novel non-destructive method to assess critical information regarding the life history of *B. occidentalis,* including that this long-extinct species could live up to 19+ years, that it was a relatively abundant faunal element, and that its populations were age heterogeneous.

##  Supplemental Information

10.7717/peerj.4819/supp-1 Supplemental Information 1 Measurements of centrum length and zygantral growth rings in *Bogertophis subocularis* and *Boavus occidentalis* (formerly YPM 2770 and 3752) Calculation of standard deviation (s) and 2s is included for *Bogertophis subocularis*. The so derived values for 2s (in %) are used to estimate whether vertebrae of *Boavus occidentalis* that are potentially from the same age are of similar size to allow assignment to the same individual.

## Additional Information and Declarations

### Data Availability

The following information was supplied regarding data availability: The Supplemental File contains the measurements and observations for the recent snakes and the fossil material.
